# The presentation order of cue and target matters in deception study

**DOI:** 10.1186/1744-9081-6-63

**Published:** 2010-10-22

**Authors:** Guangheng Dong, Yanbo Hu, Qilin Lu, Haiyan Wu

**Affiliations:** 1 Department of Psychology, Zhejiang Normal University, 688 of Yingbin Road, Jinhua City, Zhejiang Province, P.R.China; 2 Department of Psychology, Royal Holloway, University of London, Egham, UK; 3 Institute of Neuroinformatics, Dalian University of Technology. Dalian, P.R.China

## Abstract

**Background:**

Two experimental procedures (cue-target and target-cue) were used in studying the processes of deception. How the task will affect participants' performances is not clear. This study was conducted to investigate the effect of the order of presentation of cue and target on the processes of deception.

**Methods:**

A face evaluation task was employed to test and compare the order effect of the deception-indicating cue and the target stimulus in studying deception (i.e., which research procedure is more sensitive in distinguishing different experimental conditions and which is more likely to represent the deception process in daily life). Behavioral responses and event-related potentials (ERP) were recorded while participants made truthful and deceptive responses about their evaluation.

**Results:**

Response-locked ERP showed that both deceptive conditions in cue-target and target-cue procedures elicited medial frontal negativities. However, the results in the ERP distribution regions, the ERP amplitudes and source estimation results were different in the two procedures. The cue-target procedure elicited a more negative ERP deflection between 40 ms and 90 ms over the central-frontal scalp regions than the target-cue procedures. Source localizations in cue-target were identified in three clusters, namely, medial frontal gyrus, dorsal anterior cingulate cortex, and ventral medial frontal gyrus. In the target-cue procedure, the sources were identified in the frontal areas.

**Discussion:**

Different presenting orders of the cue and target stimuli induced different neural activities. Further, the cue-target procedure could represent the process of deception better than the target-cue procedure.

## Background

Various approaches to psychophysiological detection of deception have been developed recently. The polygraphic test, one of the most popular methods to detect lies by monitoring and recording peripheral measures of heart rate, skin conductance and respiration [1]. Investigators have focused on the neural basis of deception [2] and physiological measures to detect deception [3]. However, minimal knowledge on the brain mechanisms involved in the processes of deception is available [4-7]. In the past decade, brain-imaging techniques such as event related potential (ERP) and functional magnetic resonance imaging (fMRI) enable the precise recordings of brain activities that associated with the process of deception.

ERPs are widely used in studies on deception because they can provide precise temporal resolution of neural activity. An important ERP in measuring deception is the medial frontal negativities which can be elicited between 0-100 ms after a response. Medial frontal negativity amplitude is largest over the medial central-frontal scalp. This activity was initially found in conflict resolution tasks [8,9], and was labeled as the error-related negativity (ERN). However, subsequent studies have revealed that similar negativities were also elicited on correct trials [10,11], particularly when ambiguity arises in categorizing stimuli [12]. Therefore, some investigators suggested that error related negativity could represent the activity in neural circuits which are responsible for the executive processes, such as correcting an error and/or monitoring the participant's actions, thus, they name it medial frontal negativity [8,10].

Localization studies have placed the neural generators of the error related negativity and medial frontal negativity in different locations within the medial frontal lobes, in or near the anterior cingulate cortex [1,13-15]. The fMRI results showed that the source of the medial frontal negativity has been located more caudally in the anterior cingulate cortex than the source of the error related negativity [16,17]. Hence, these localizations suggest that the error related negativity and the medial frontal negativity may represent the neurophysiological activity implicated in the cited fMRI studies. A one-to-one relation is noted between ERP and hemodynamic results due to different aspects of brain activity as recorded by the two measures. Given that the relationship between the negativities elicited is not resolved through correct and error trials, we used Gehring and Willoughby's [10] term, medial frontal negativity, to refer to this activity when it is elicited on correct trials.

Some investigators have found that the medial frontal negativity reflects activity in anterior cingulate cortex, a brain area involved in monitoring actions and resolving conflicting response tendencies [1,10,18]. The medial frontal negativity has been linked to response monitoring and the degree of response conflict created by a stimulus [11,14,19]. Results from Johnson's study showed that the deceptive responses elicited significantly larger medial frontal negativity compared with that of the truthful responses, which indicated that the medial frontal negativity might be involved in response monitoring and conflict detection [1]. The medial frontal negativity elicited by the directed and self-generated lies were due to different patterns of brain activity respectively, and were both different from that of truthful medial frontal negativity [16].

To study the cognitive process of deception, the researchers categorized the general types of processes that might be used by a deceptive answer. Although deception does not specify the cognitive processes involved, its process can be divided into two broad stages: (1) the cognitive/emotional processes used to formulate factors such as intent and strategies relevant to a deception; and (2) those used in the act of deception [20]. The task procedures used in this study are equivalent to the above mentioned process of deception. First, participants see the cues and decide that they should make a deceptive answer; subsequently, they carry out the deceptive action required. Therefore, this research procedure can properly represent the process of deception.

**Figure 1:**
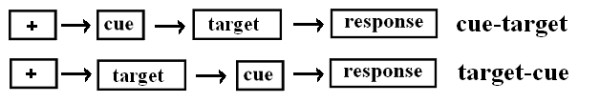
Different experimental procedures in deceptive study

In a standard research procedure of deceptive processes, participants were asked to press one button upon seeing an old word and the other button for a new word. In deceptive condition, they were then told 'to lie or try to hide what they know by intentionally pressing the answer opposite the instructions' [16,21]. Whether the participants should respond truthfully or deceptively relied on the cue stimuli. There are two variations for target presentation of the paradigm: the cue stimuli were shown before the target stimuli (cue-target) and the cue stimuli were presented after the target stimuli (target-cue) (Figure 1). ERP has a high time resolution and is sensitive to the slight changes between the mental processes which have advantages in detecting the order effect in different tasks. Although both target-cue and cue-target procedures were used in measuring deception, how the task order will affect participants' performances remained uncertain. In the cue-target procedure, participants know whether they should make deceptive responses first, and then, provide judgments about the stimuli. It is a standard 'evaluate-deceptive' procedure. On the other hand, in the target-cue procedure, participants make judgments about the target stimuli first, after which, the cue stimuli suggest whether or not they should make deceptive responses. It is looks like a 'coordination' process in conflicting or mismatch situation. Comparing the validity of the two procedures on deception study is necessary. The numerous research paradigms in this field may bring about varied results. Hence, drawing the comparisons between paradigms is important in order to enhance understanding of their advantages and disadvantages. In addition, the validity of existing research paradigms on deception was often questioned by researchers. Therefore, we need to explore the cognitive processes of the deception to show its details. The two procedures, namely, cue-target and target-cue, were by compared through the features of medial frontal negativity.

## Methods

### Participants

Nineteen right-handed (determined by Henkel's digitizing tablet method [22]) subjects participated in this experiment (10 female,9 male), all of whom had normal or corrected to normal vision and did not have any history of neurological disease. Data from three female subjects were discarded because of too many artifacts. The valid subjects were 18.6 to 26.4 years old (mean age: 22.3 years). The experiment procedure was in accordance with the ethical principle of the 1964 Declaration of Helsinki (World Medical Organization).

### Materials

Facial pictures were chosen as stimuli and were delivered through E-Prime software (Version 1.2) (Psychology Software Tools Inc., Pittsburgh, Pennsylvania, USA). All stimuli pictures were taken from the Internet. In order to avoid the fondness for special people, no celebrities such as movie stars or politicians were selected. All of the stimuli pictures were selected from ordinary people and were unfamiliar with the participants. The emotional expressions of people in the pictures were neutral (28 university students were asked to evaluate the expression of the facial pictures (positive, negative, and neutral). Only those neural pictures with no disaccords were selected). All the stimuli pictures measured 360 (width) × 480 (length) pixels (when running the E-Prime software, the whole screen measures 640 × 480 pixels). The pictures showed the front part of the face and at least two thirds of the entire picture was the face. All the pictures were colored gray with a black background.

To select the facial stimuli, 28 college students were asked to rate the valence of 300 pictures (valence: attractive vs. ugly; arousal level) by self-report using a five-point rating scale before formal study. Based on their rating results, 60 attractive (30 men, 30 women) and 60 unattractive facial pictures (30 men, 30 women) were selected as stimuli materials in our study. These two types of pictures showed a significant difference in valence values (attractive: 3.87 ± 0.68; unattractive: 1.35 ± 0.56) [*F*(1,27) = 6.601, *p *< 0.01]. The arousal levels were about the same (attractive: 2.87 ± 0.64; unattractive: 2.75 ± 0.59). All pictures were presented in both of these two blocks. There are 60 trials for each condition. As the 'evaluation' is a subjective judgment process, and the personal viewpoints may disturb the final results, we excluded the answers that were not agreed with defaulted values.

### Tasks and procedures

Subjects were seated in a quiet room, approximately 80 cm away from a computer screen (DELL, 17-inch LCD monitor, 60-Hz refresh rate) with less than 5° of visual angle in both horizontal and vertical directions. All subjects were required to fixate at the screen during all tasks.

The study was divided into three steps. In the first one, the pilot process, each trial started with a small white cross (+) at the center of the screen against a black background for 250 ms followed by a stimulus picture shown for 1000 ms. Participants were instructed to press relevant keys with the fingers they used mostly in their right hand when the stimuli disappeared (attractive, 1; unattractive, 2). A 500 ms black screen would appear as inter-trail interval after a response was made. The main purpose of this step was to familiarize participants with the task procedure. Data from step 1 were not included in future analyses.

In step 2, each trial started with a small white cross (+) in the center of the screen against a black background for 250 ms. Then, a cue word 'T' for 'truthful' and 'D' for 'deceptive' was presented randomly in the center of the screen for 1000 ms (before the experiment, participants were informed about the meaning of T and D). The stimulus picture was presented for 1000 ms after the instructive cue. Participants were instructed to make truthful 'attractive or unattractive' judgments about the pictures when the cue word was 'T' and were required to press key '1' for attractive pictures and key '2' for unattractive pictures. In deceptive conditions, participants were required to make judgments about the pictures and give the opposite responses when the cue word was 'D'. In fact, they were told to lie or to try hiding their real evaluation by doing exactly the opposite reaction after the stimuli disappeared. In step 3, the procedures were of the same with step 2 except the target stimuli were presented first, and the cue words were presented after the target stimuli.

Step 2 and 3 consisted of two blocks each and were tested in ABBA order to counterbalance potential testing order effect. In the A section, participants perform cue-target procedure, in the B section, participant perform target-cue task first. Each subject participated in all of these three steps, with counter-balanced order of the latter two steps between subjects (ABBA, BAAB).

### ERP recording

High-density ERPs were recorded from each participant using a 128-channel geodesic sensor net (Electrical Geodesics Inc., (EGI) Eugene, Oregon, USA) coupled with a high input impedance amplifier. The EEG was continuously recorded at a sample rate of 250 Hz. Whenever possible, impedances were reduced to less than 50 KΩ prior to recording with the vertical electrooculograms (EOG) recorded at the left orbital rim and the horizontal EOG recorded at the right orbital rim.

### ERP averaging

The data were analyzed offline with the software NetStation (Electrical Geodesics Inc., Eugene, Oregon, USA). Trials with incorrect responses (responses different with defaulted value) and trials with EOG artifacts (>50 μV) were discarded. In present study, responses that were not agreed with defaulted value were thought incorrect. Such as there is an attractive face, the cue is 'deceptive', and then the right answer should be 'unattractive'. However, if participants answered 'attractive', that answer is incorrect. The data were filtered with a band pass of 0.3-30 Hz. EEG activity for the correct response in each valence condition was overlapped and pre-processed (filter, epoch, artifact detection, bad channel replacement, average reference, separate average, baseline correction) separately. The ERP waveforms were averagely referenced and response-locked (0 ms is the time point when participants made response). The average epoch was 400 ms, including a 200 ms pre-response baseline. The percentage of rejected epochs in each condition was less than 25%. If the rejected epochs exceeded that number, data of this participant would be excluded from further analysis.

Localization studies have consistently placed the neural generator of the error related negativity in the medial frontal lobes, in or near the anterior cingulate cortex [23,24]. Additionally, topographic maps showed the anterior cingulate cortex were activated during this process. So, in our study, we selected F3, Fz, F4, FC3, FCz, FC4, (6 frontal sites), C3, Cz, C4 (3 central sites) for analysis. Repeated ANOVAs were conducted to test the difference between different procedures (cue-target, target-cue) in medial frontal negativity (40-90 ms) components. Because the data from multiple electrode sites may lead to a violation of the sphericity assumption, Bonferroni correction was applied for multiple post-hoc (LSD) comparisons when appropriate.

### Source estimation

Source estimates of the scalp potential were accomplished using the GeoSource electrical source imaging software (EGI, Eugene, OR). GeoSource used a finite difference model (FDM) for the accurate computation of the leading field in relation to cranial orifices (primarily optical canals and foramen magnum). Conductivity values that used in the FDM model were as follow: 0.25 S/m (Siemens/meter) for brain, 1.8 S/m for cerebral spinal fluid,0.018 S/m for skull, and 0.44 S/m for scalp [25]. Source locations were derived from the Montreal Neurological Institute probabilistic MRI. Once the head model was constructed, an average of the 128-channel positions was registered to the scalp surface. To compute the estimates of the sources, a minimum norm solution with the LAURA (local autoregressive average) constraint [26] was employed.

## Results

### Behavioral performance

**Figure 2:**
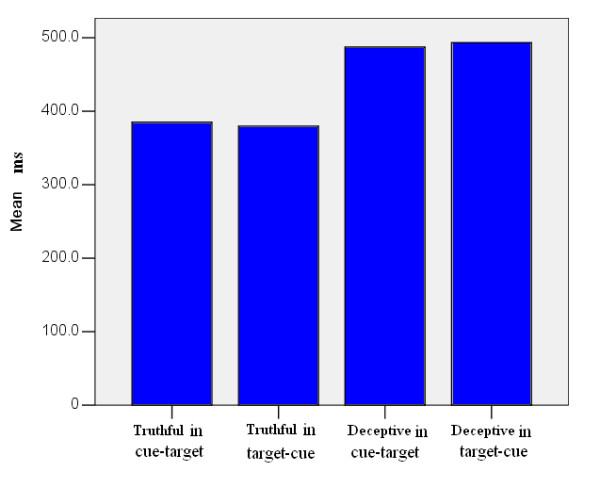
**Reaction times in different conditions**. Figure 2 show the reaction times in truthful and deceptive conditions in cue-target and target-cue procedures.

Responses that were too fast (less than 100 ms), too slow (more than 1000 ms), and incorrect responses were excluded from the final analysis. The mean reaction times (RT) of the deceptive responses for the Truthful trials in cue-target, Truthful trials in target-cue, Deceptive trials in cue-target, and Deceptive trials in target-cue were 385.1 ms (*SD *= 116.5), 379.7 ms (*SD *= 114.9),487.3 ms (*SD *= 149.7) and 493.2 ms (*SD *= 156.9), respectively. The RT in Truthful conditions were significant shorter than in deceptive conditions (cue-target [*F*(2,30) = 4.933, *p *< 0.05], target-cue [*F*(2,30) = 5.025, *p *< 0.05]). No significant difference was found between target-cue and cue-target procedures [*F*(2,30) = 0.902, *p *> 0.05] (Figure 2).

### ERP results

In the cue-target procedure, the medial frontal negativity in deceptive condition showed significant higher mean amplitude than that in truthful condition [*F*(2,30) = 5.193, *p *< 0.05]; this feature was also found between deceptive and truthful conditions in target-cue procedure [*F*(2,30) = 4.653, *p *< 0.05]. No significant difference of peak latencies was found between deceptive and truthful conditions in cue-target [*F*(2,30) = 1.127, *p *> 0.05] and target-cue [*F*(2,30) = 0.795, *p *> 0.05] procedures. These results showed consistency with previous results about the features of medial frontal negativity between deceptive and truthful conditions [1,10,18]. Further analysis showed that the distribution were different between cue-target and target-cue in deceptive conditions. In cue-target procedure, all sites showed significant higher amplitude for the Deceptive than the Truthful condition. However, only frontal sites (F3, Fz, F4, FC3, FCz, FC4) were showed significant difference between target-cue (deceptive) and truthful conditions. No significant difference was found in central sites between them.

**Figure 3:**
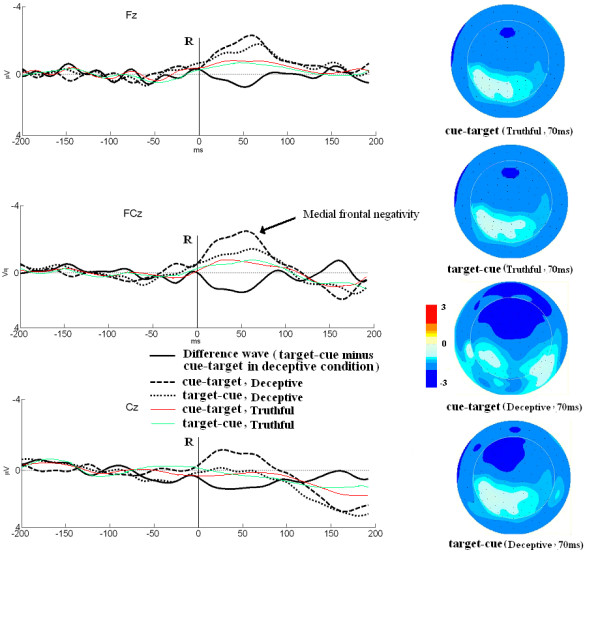
Grand averaged waveforms at Fz, FCz, and Cz in different procedures (Left); Topographical maps in different procedures at 70 ms after response (Right)

ERP waveforms of deceptive conditions in both cue-target and target-cue procedures showed distinct medial frontal negativity components. The cue-target procedure elicited a more negative ERP deflection between 40 ms and 90 ms over the central-frontal scalp regions than target-cue procedures (Figure 3). A significant main effect was found between cue-target and target-cue deceptive procedures in mean amplitude [*F*(2,30) = 9.922, *p *< 0.01]. Peak latencies were also compared between deceptive conditions in cue-target and target-cue procedures, however, no significant difference was found in peak latencies between these two procedures [*F*(2,30) = 1.021, *p *> 0.05].

### Source estimation results

**Figure 4:**
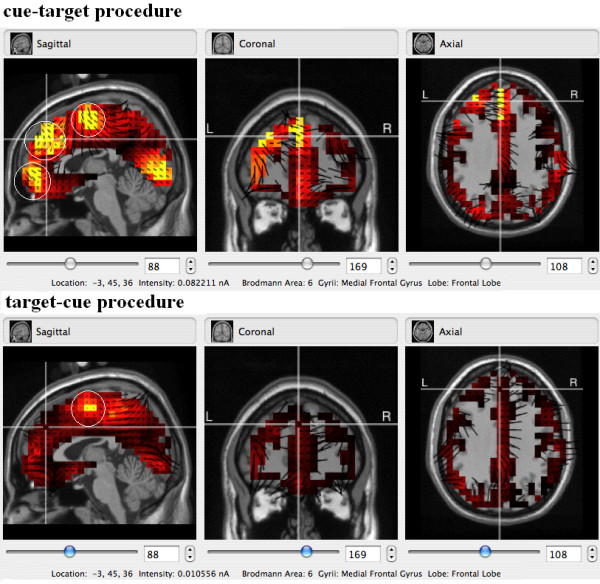
**Source estimates for medial frontal negativity in 70 ms after response in different deceptive procedures**. In this figure, brighter colors denote stronger current sources. The figure shows the source estimation results for medial frontal negativity in target-cue and cue-target procedures in 70 ms (peak of medial frontal negativity) after response.

The estimated source regions contributing to the medial frontal negativity, at its peak, were illustrated in Figure 4. For the medial frontal negativity that associated with the cue-target procedure in the post-response state, sources were identified in three clusters: (1) medial frontal gyrus (Brodmann area (BA) 6 and 8), (2) dorsal anterior cingulate cortex (BA 24 and 32) and (3) ventral medial frontal gyrus (BA 10), orbital gyrus (BA 11). In comparison, for the target-cue procedure, sources were identified in only one clusters, which located in frontal areas (about near anterior cingulate cortex area). The difference between these two deceptive procedures can be found in activity levels and the related source areas (Figure 4).

## Discussion

Face evaluation task was employed to test the order effects of the deception-indicating cue and the target stimulus with a response-locked experimental paradigm. The task has advantages over the previous paradigms. First, it is not memory-based response conflicts; such that, the participants need only to respond according to their current judgment. Second, deception is usually accompanied by an emotion experience, and deception in terms of assessing attractiveness/unattractiveness is most likely to occur in real life [7].

Behavioral data in RT showed that deceptive conditions were more difficult (longer RT) than truthful conditions in relation to presenting orders. The increased behavioral cost of making directed lies about the evaluation can be found with the greater RT costs. Therefore, conflict effect can be observed clearly. The results were consistent with previous findings on deceptive processes [1,10,18]. In addition, no significant difference was found between the deceptive conditions in cue-target and target-cue procedures; but the difference between these two conditions was found in ERP results.

For ERP results, deceptive condition showed higher medial frontal negativity mean amplitudes than truthful condition in both target-cue and cue-target procedures. This indicates that these two deceptive research procedures have significant similarity during the conduct of the processes in ERP waveforms. The ERP features were consistent with previous results about medial frontal negativity [1]. Further analysis showed the difference between the procedures: the distribution regions of this significant effect were different. In the deceptive condition of cue-target procedure, the significant effect can be found over the frontal-central sites. This is in accordance with previous studies on the distribution of the medial frontal negativity using other deceptive processes [27]. However, only frontal sites showed significant effect in the deceptive condition of target-cue procedure; no significant difference was found in central sites.

Medial frontal negativity was observed at 40-90 ms after the response in deceptive conditions in both cue-target and target-cue procedures. The comparison results between the valences indicate that the deceptive condition in cue-target procedure elicited a more negative ERP deflection than in target-cue procedure in the central-frontal area. Results were consistent with previous studies in which the deceptive responses elicited significantly larger medial frontal negativity compared to the truthful responses [1,10,18]. The medial frontal negativity component has been linked to response monitoring and the degree of response conflict created by a stimulus [10,11,18,28,29]. The medial frontal negativity was believed to reflect activity in anterior cingulate cortex, a brain area involved in monitoring actions and resolving conflicting response tendencies [1,16]. Therefore, the higher medial frontal negativity in the deceptive condition in cue-target procedure compared with the deceptive condition in target-cue procedure indicates that the participants engaged more attention resource in monitoring and detecting conflict situations.

The estimated source regions contributing to different deception procedures, at their peaks, are illustrated in Figure 4. In the deceptive condition of cue-target procedure, sources were identified in three clusters;, while in target-cue procedures, only one cluster was found. Previous mapping studies have reported the activation of discrete anterior frontal regions during deception. These regions are the ventrolateral prefrontal cortex, dorsolateral prefrontal cortex, dorsal medial prefrontal cortex, and anterior cingulated cortex during deception [30]. The analyses found that the source estimate of the deceptive condition in cue-target procedure is more consistent with the fMRI results of deception compared with of the target-cue procedure.

Results of ERP waveforms (broader distribution, more similarities with previous results, higher amplitude) and the source estimation (more activation areas) showed that the cue-target procedure was better than the target-cue procedure in measuring deceptive processes. This is a result of the sequence of the mental processes that caused by the sequence of presentation of cue and target in different deceptive procedures. The sequence of mental processes distinguishes one procedure from another. In the cue-target procedure, participants knew beforehand whether they should make a deceptive response according to the prior cue. When the target stimulus was presented, they immediately evaluated the feature of target while trying to suppress their real thoughts. The mental process 'evaluate-deceptive' was similar to the real deceptive process in daily life (deceptive idea is conceived first prior to performing it). On the other hand, in the target-cue procedure, participants evaluated the pictures first when the target stimulus was presented. Subsequently, they coordinated their previous judgment and responded according to the following cue stimulus. The mental process 'coordination' serves as an executive control process in regulating our thoughts and behaviors (the conflicting or mismatch process) [31]. The deceptive process is comparable with conflicting process.

The medial frontal negativity results indicate that the anterior cingulate cortex plays an important role in both controlling and monitoring a person's actions or decisions when conflicting information arise while determining which response is correct [32-34]. Previous studies showed that the brain activity associated with response conflicts, that occurred during the execution of deceptive responses, was independent of the source of the conflicting response information; accordingly, both perceptually- and memory-based conflicts appear to enlist the activity of the same medial frontal brain circuits [16]. Similar to previous results [35,36], these response conflicts were processed by patterns of brain activity that differed from those used to process conflicts regarding proper stimulus categorization (i.e., the old-new differences). Therefore, ERP features in conflicting process were similar with those in deceptive process; the cue-target procedure was more consistent with the real deceptive procedure, while the target-cue procedure in measuring deception served as an executive process in the minds of the participants. In summary, different mental processes elicited brain activities during experimental procedures. This explains the difference between these two procedures.

### Limitations and future directions

Some limitations of the present study should be noted. First, during the intending process, controlling the participant's strategy use was difficult. Future studies should therefore determine other methods to address this issue. Secondly, all participants were right handed students. However, handedness may or may not affect the mental process. As such, this should be a focus of future research. Thirdly, other variables may affect the results in target-cue procedures. Further studies should specify the different processes involved in target-cue procedure.

## Conclusions

The different presenting orders between cue and target stimuli brought about different mental processes. These processes induced different neural activities. In studying deception, the cue-target procedure was found to be more effective than the target-cue procedure.

## List of abbreviations

ACC: Anterior cingulate cortex; ANOVA: Analyses of variance; EEG: electroencephalogram; EOG: Electrooculograms; ER: Error rate; ERN: Error-related negativity; ERP: Event-Related potential; fMRI: Functional magnetic resourcing imaging; MFN: Medial frontal negativity; RT: Reaction time; SD: Standard deviation.

## Competing interests

The authors declare that they have no competing interests.

## Authors' contributions

GD carried out the study. YH participated in the design of the study. QL participated in the paper writing and correction. HW performed the statistical analysis. All authors read and approved the final manuscript.
