# The presentation order of cue and target matters in deception study

**DOI:** 10.1186/1744-9081-7-36

**Published:** 2011-08-16

**Authors:** Guangheng Dong, Yanbo Hu, Haiyan Wu

**Affiliations:** 1 Department of Psychology, Zhejiang Normal University, 688 of Yingbin Road, Jinhua City, Zhejiang Province, P.R.China; 2 Department of Psychology, Royal Holloway, University of London, Egham, UK

## 

The original article [1] was initially published with the following list of authors: Guangheng Dong, Yanbo Hu, Qilin Lu and Haiyan Wu. This author list is now corrected as follows: Guangheng Dong, Yanbo Hu and Haiyan Wu. As Qilin Lu's contribution in writing the article did not fulfill the authorship criteria of being involved in drafting the manuscript for important intellectual content he has been removed from the author list. As such the authors' contributions section has been amended accordingly.

## Authors' contributions

GD carried out the study. YH participated in the design of the study. HW performed the statistical analysis. All authors read and approved the final manuscript.
